# Effect of dentate gyrus disruption on remembering what happened where

**DOI:** 10.3389/fnbeh.2015.00170

**Published:** 2015-06-30

**Authors:** Woon Ryoung Kim, Jong Won Lee, Woong Sun, Sung-Hyun Lee, June-Seek Choi, Min Whan Jung

**Affiliations:** ^1^Department of Anatomy, College of Medicine, Korea UniversitySeoul, Korea; ^2^Center for Synaptic Brain Dysfunctions, Institute for Basic ScienceDaejeon, Korea; ^3^Neuroscience Graduate Program, Ajou University School of MedicineSuwon, Korea; ^4^Department of Psychology, Korea UniversitySeoul, Korea; ^5^Department of Biological Sciences, Korea Advanced Institute of Science and TechnologyDaejeon, Korea

**Keywords:** hippocampus, episodic memory, working memory, bax, delayed response task

## Abstract

Our previous studies using Bax knockout (Bax-KO) mice, in which newly generated granule cells continue to accumulate, disrupting neural circuitry specifically in the dentate gyrus (DG), suggest the involvement of the DG in binding the internally-generated spatial map with sensory information on external landmarks (spatial map-object association) in forming a distinct spatial context for each environment. In order to test whether the DG is also involved in binding the internal spatial map with sensory information on external events (spatial map-event association), we tested the behavior of Bax-KO mice in a delayed-non-match-to-place task. Performance of Bax-KO mice was indistinguishable from that of wild-type mice as long as there was no interruption during the delay period (tested up to 5 min), suggesting that on-line maintenance of working memory is intact in Bax-KO mice. However, Bax-KO mice showed profound performance deficits when they were removed from the maze during the delay period (interruption condition) with a sufficiently long (65 s) delay, suggesting that episodic memory was impaired in Bax-KO mice. Together with previous findings, these results suggest the role of the DG in binding spatial information derived from dead reckoning and nonspatial information, such as external objects and events, in the process of encoding episodic memory.

## Introduction

Hippocampus plays an essential role in encoding episodic (or episodic-like) memory (Scoville and Milner, 1957; Nadel and Moscovitch, 1997; Eichenbaum et al., 1999; Squire et al., 2004), which is the memory of a specific past event that occurred at a particular time and place (Morris, 2001; Clayton et al., 2001; Dere et al., 2006; Ferbinteanu et al., 2006; Eacott and Easton, 2010; Eichenbaum et al., 2012). Hippocampal neurons in rodents show strong location-specific discharges (O’Keefe and Dostrovsky, 1971; Jung and McNaughton, 1993), indicating that allocentric spatial information is represented in the hippocampus. Hippocampal neuronal activity is also modulated by various nonspatial factors, such as odor (Wood et al., 1999; Deshmukh and Bhalla, 2003), navigation mode (Song et al., 2005; Ravassard et al., 2013), reward (Kobayashi et al., 1997; Hölscher et al., 2003; Smith and Mizumori, 2006), punishment (Segal et al., 1972; Berger et al., 1976; McEchron and Disterhoft, 1997; Múnera et al., 2001; Moita et al., 2003, 2004), value (Lee et al., 2012a), movement trajectory (Frank et al., 2000; Wood et al., 2000), history of past choices and outcomes (Eichenbaum et al., 1987; Lee et al., 2012a), and elapsed time (Itskov et al., 2011; MacDonald et al., 2011; Naya and Suzuki, 2011), indicating that the hippocampus conjunctively conveys spatial and nonspatial information that are building blocks of episodic memory.

Although the hippocampal role in encoding episodic memory is well established, it is unclear how spatial and nonspatial information are integrated in the hippocampus. Entorhinal cortex (EC), which consists of medial and lateral divisions, provides major inputs to the hippocampus (van Strien et al., 2009). Characteristics of grid cells found in the medial EC (Hafting et al., 2005; Sargolini et al., 2006) suggest representation of the internally-generated spatial map (i.e., spatial map generated by dead reckoning) in this brain structure (Leutgeb et al., 2005; O’Keefe and Burgess, 2005; Fuhs and Touretzky, 2006; McNaughton et al., 2006; Witter and Moser, 2006; Gorchetchnikov and Grossberg, 2007). By contrast, neurons in the lateral EC show little spatially-selective, but object-dependent firing (Hargreaves et al., 2005; Yoganarasimha et al., 2011), suggesting segregation of spatial and nonspatial information processing in the medial and lateral EC, respectively (Knierim et al., 2006; Eichenbaum et al., 2007; Kerr et al., 2007; Deshmukh and Knierim, 2011). Because inputs from the medial and lateral EC converge in the dentate gyrus (DG) and CA3, it has been proposed that the internally-generated spatial map is associated with external landmarks in the DG-CA3 network, forming a distinct spatial context for each environment (Redish and Touretzky, 1997; Hafting et al., 2005; O’Keefe and Burgess, 2005; Knierim et al., 2006, 2014; Witter and Moser, 2006; Gorchetchnikov and Grossberg, 2007; Leutgeb and Leutgeb, 2007).

In this regard, we have shown previously that hippocampal spatial firing is dissociated from external landmarks in Bax knockout (Bax-KO) mice (Lee et al., 2009, 2012b), in which newly generated granule cells continue to accumulate, disrupting neural circuitry specifically in the DG (Sun et al., 2004; Kim et al., 2009). Behaviorally, Bax-KO mice were impaired in finding a target location based on visual landmarks when target locations predicted by dead reckoning and visual landmarks were made incongruent (Lee et al., 2009). These results suggest the involvement of the DG in binding the animal’s internal spatial map with the sensory information on external landmarks in forming a distinct spatial context for each environment. However, considering that the EC provides major cortical inputs to the hippocampus (van Strien et al., 2009), it is likely that the DG plays a more general role in encoding episodic memory than merely forming a distinct spatial context for each environment (Kesner, 2007). In the present study, we examined whether intact DG is necessary for remembering “what *happened* where” (i.e., spatial map-event association) in addition to remembering “what* is* where” (i.e., spatial map-object association) using a delayed-non-match-to-place task. We obtained results that are consistent with a general role of the DG in binding spatial and nonspatial information in the process of forming episodic memory.

## Materials and Methods

### Subjects

Five Bax-KO mice and five wild-type littermates (C57BL/6J genetic background) were used in the present study. Homozygous Bax-KO and wild-type littermate mice were obtained by crossing heterozygous males and females as previously described (Sun et al., 2004). All animals were water-deprived with free access to food (maintained >80% of ad libitum body weight) and handled extensively before behavioral training began. They were maintained at a 12-h light/dark cycle and performed the behavioral task in the dark phase. The experimental protocol was approved by the Institutional Animal Care and Use Committee of the College of Medicine, Korea University.

### Behavioral Task

**Figure 1:**
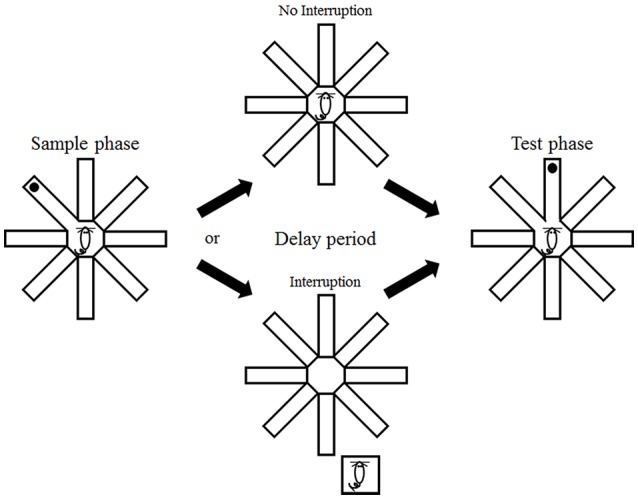
**Behavioral task.** The animals were tested in a delayed non-match-to-place task in an eight-arm radial maze. They were forced to visit one of the eight arms (sample phase) that was presented with a randomly-chosen adjacent arm (test phase) with a delay. The animals either remained in the maze (no interruption condition) or were removed from the maze (interruption condition) during the delay period.

The animals were tested in a delayed-non-match-to-place task in an open eight-arm radial maze (central platform, diameter: 20 cm; arms, length: 35 cm, width: 5 cm; height of walls along the arms: 20 cm) that was placed near one corner of the testing room (3 × 4 m) containing rich visual cues. A trial began by placing an animal at the central platform and opening the door of a randomly chosen arm (sample arm) with all of the other doors closed. The animal was rewarded with 15 μl of water at the end of the sample arm (sample phase). The door of the sample arm was closed when the animal came back to the central platform, which marked the beginning of a delay period. The animal was either allowed to stay in the maze during the delay period (no interruption condition) or placed in its home cage (located ~50 cm away from the maze; the same location throughout the experiment), and then placed back on the central platform immediately before the end of the delay period (interruption condition). The doors of the sample arm and a randomly-chosen adjacent arm were open at the end of the delay period (test phase; Figure 1). The animal was rewarded with 15 μl of water by visiting an arm other than the sample arm. Each daily session consisted of a total of six trials with 2 min inter-trial intervals.

### Statistical Analysis

Two-way ANOVA (repeated measure), linear regression analysis, and Student’s *t*-tests (two-tailed) were used for statistical comparisons of the animal’s performance. A *p* value < 0.05 was used as the criterion for a significant statistical difference. All data are expressed as mean ± S.E.M.

## Results

**Figure 2:**
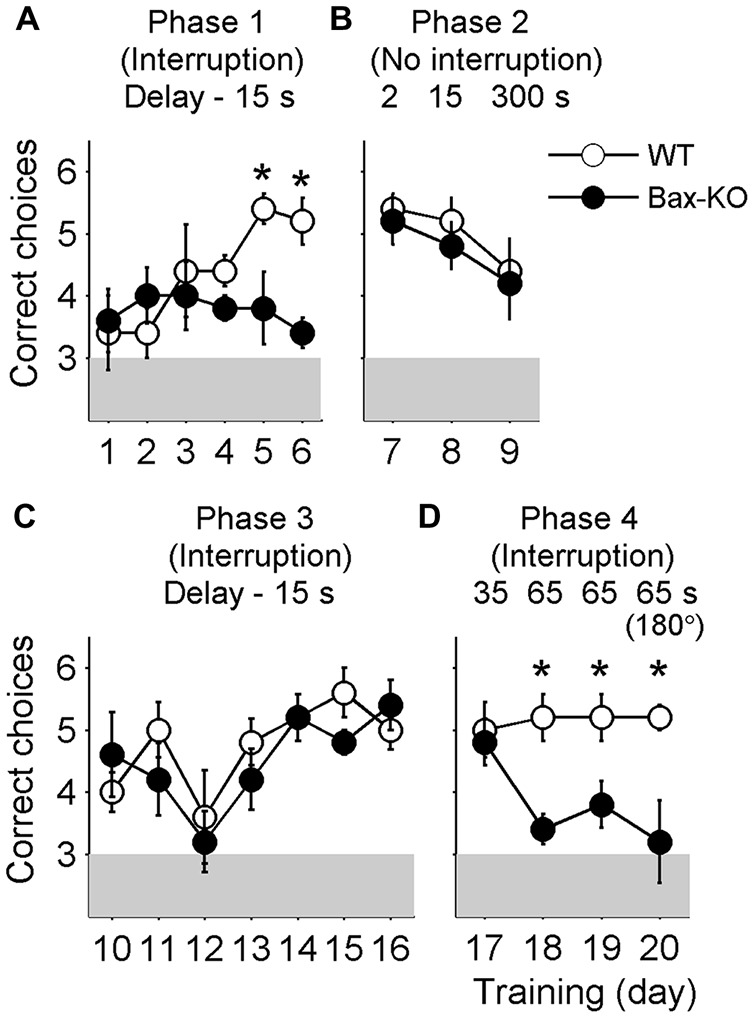
**Behavioral performance.** The animals went through four different phases of training during 20 days. Shown are mean numbers of correct choices per daily session (out of a total of six trials per daily session) during phase 1 (**A**, interruption condition, delay duration = 15 s, days 1–6), phase 2 (**B**, no interruption condition, delay duration = 2, 15 and 300 s, days 7–9), phase 3 (**C**, interruption condition, delay duration = 15 s, days 10–16), and phase 4 training (**D**, interruption condition, delay duration = 35 and 65 s, days 17–20). On day 20 (the last day of training) the animals were tested with two opposite arms (indicated as 180°) instead of two adjacent arms. *, significant difference between animal groups (*p* < 0.05).

The animals were initially trained with a 15 s delay under the interruption condition (i.e., they were removed from the maze during the delay period; phase 1 training). The number of correct choices gradually increased over 6 days of training in wild-type littermates (linear regression analysis, slope = 0.429, *t*-test, *p* = 4.9 × 10^−4^), but not Bax-KO mice (slope = −0.051, *p* = 0.616; Figure 2A). Two-way ANOVA indicated a trend for significant group × day interaction (main effects of the animal group: *F*_(1,8)_ = 3.411, *p* = 0.102; training day: *F*_(5,40)_ = 1.706, *p* = 0.155; group × day interaction: *F*_(5,40)_ = 2.392, *p* = 0.055), and *post hoc* Bonferroni comparison indicated significant differences between wild-type and Bax-KO mice on days five (*p* = 0.035) and six (*p* = 0.004).

When the delay duration was reduced to 2 s with no interruption (i.e., the animals were allowed to stay in the maze; phase 2 training), both groups performed well. As the delay duration increased to 15 s and then to 5 min, Bax-KO mice performed comparably to the control group (2 s delay: *t*_(8)_ = 0.447, *p* = 0.667; 15 s delay: *t*_(8)_ = 0.756, *p* = 0.471; 5 min delay: *t*_(8)_ = 0.258, *p* = 0.803), although the level of performance dropped progressively as the delay duration increased (Figure 2B). These results show that Bax-KO mice, as long as there was no interruption, could retain working memory of a previously visited location for a relatively long period of time.

When the animals were re-trained with a 15 s delay with the removal from the maze during the delay (interruption condition; phase 3 training), both wild-type littermates and Bax-KO mice showed significant enhancement of behavioral performance (the number of correct choices) over 7 days of training (linear regression analysis, wild-type littermates, slope = 0.207, *t*-test, *p* = 0.026; Bax-KO mice, slope = 0.200, *p* = 0.046). Two-way ANOVA also indicated a significant main effect of training day (*F*_(6,48)_ = 5.140, *p* = 3.8 × 10^−4^) without a significant main effect of animal group (*F*_(1,8)_ = 0.371, *p* = 0.559) or group × day interaction (*F*_(6,48)_ = 0.961, *p* = 0.462; Figure 2C). These results suggest that the initial learning deficit of Bax-KO mice was likely due to a problem in representing the relationship between actions and outcomes or learning a task rule.

This pattern persisted up to 35 s delay (phase 4 training; comparison between Bax-KO and wild-type mice, day 17, *t*_(8)_ = 0.343, *p* = 0.740, Figure 2D). However, as the delay duration increased to 65 s (animals removed from the maze), the performance of Bax-KO mice was dramatically impaired compared to the wild-type littermates (day 18, *t*_(8)_ = 4.025, *p* = 0.004; day 19, *t*_(8)_ = 2.646, *p* = 0.029, Figure 2D). The impairment at 65 s delay persisted even when the correct arm was the opposite of the sample arm, instead of an adjacent arm (day 20, *t*_(8)_ = 2.887, *p* = 0.020, Figure 2D).

## Discussion

**Figure 3:**
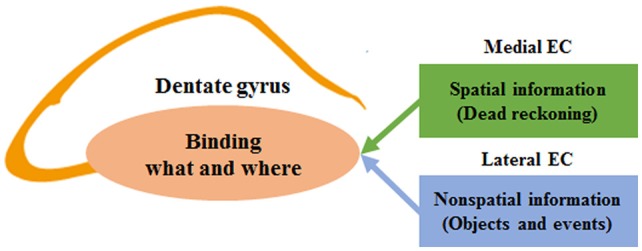
**A model for the role of the dentate gyrus (DG) in binding spatial and nonspatial information in encoding episodic memory.** The schematic diagram shows simplified functional connections between the entorhinal cortex (EC) and DG. The DG receives converging inputs from the medial and lateral EC. The medial EC carries spatial information derived from dead reckoning (internally-generated spatial map), and the lateral EC carries nonspatial information (sensory information on external objects and events). The two streams of information are combined in the DG in the process of encoding episodic memory.

We have shown previously that spatial firing of hippocampal neurons in Bax-KO mice was dissociated from an external landmark, and that Bax-KO mice followed dead reckoning instead of landmarks when there was a mismatch between dead reckoning- and landmark-based prediction of a goal location (Lee et al., 2009, 2012b). These results provide empirical evidence for the involvement of the DG in aligning the internally-generated spatial map to external landmarks. The present study shows that Bax-KO mice are impaired not only in using external sensory stimuli, but also in remembering a previously visited location in navigating toward a rewarding location. Performance of Bax-KO mice was intact even with a long (up to 5 min) delay, as long as there was no interruption during the delay. However, Bax-KO mice were profoundly impaired when the delay was sufficiently long (65 s) and there was an interruption during the delay, suggesting that the nature of the deficit is not the inability to hold information on-line as working memory, but more likely to be impaired episodic memory. These results suggest a broader role of the DG in associating spatial (“where”) and nonspatial information (objects and events; information on “what”) that are required for encoding episodic memory (Figure 3). A recent study that created false fear memory by activating neutral context-associated DG neurons during fear conditioning in a different context (Ramirez et al., 2013) is also consistent with the role of the DG in binding spatial and nonspatial information. In addition, context learning is correlated with synaptic plasticity in the EC-DG pathway, which has a different time course from synaptic plasticity in intrahippocampal circuits during associative learning (Gruart et al., 2014; Carretero-Guillén et al., 2015). Future studies should investigate whether the DG is generally involved in binding spatial and nonspatial information across different experimental settings.

A previous study tested the behavioral performance of mice lacking an N-methyl-D-aspartate (NMDA) receptor subunit in the DG in a six-arm radial maze (Niewoehner et al., 2007). In this study, three arms were always baited, and the other three were never baited. Thus, the animals had to remember which three arms to visit to obtain rewards (spatial reference memory). In addition, in each trial, the animals had to remember which arm to visit next (spatial working memory). On the one hand, the NMDA-receptor mutant mice showed intact reference memory, which is consistent with our previous findings that Bax-KO mice showed intact performance in spatial reference memory tasks as long as there was no mismatch between dead reckoning and visual landmarks (Lee et al., 2009). On the other hand, the NMDA-receptor mutant mice were impaired in the working memory component of the task. It is difficult to directly compare this result with ours because the task structures are different. It is worth noting, however, that this task is similar to the interruption condition of our task in that the mice had to remember multiple visited locations with intervening behavior. Previous studies have also shown that the hippocampus is required for intact performance in multiple-location spatial working memory tasks (Olton et al., 1979; Murray et al., 2011). Thus, neural processes supporting on-line working memory, such as persistent neural activity (Fuster and Alexander, 1971; Kubota and Niki, 1971; Funahashi et al., 1989), might be insufficient to keep track of multiple locations that the animal has visited. Instead, it is more likely that the episodic memory function of the hippocampus is required to solve the three-arm working memory task. Although there exists uncertainty regarding the nature of impaired neural processes underlying impaired performance in a spatial working memory task, these and our results are in line with the requirement of the DG in forming memories of “what happened where”.

Because the medial and lateral EC send converging projections to the DG and CA3, it has been proposed that spatial and nonspatial information are combined in the DG-CA3 network (Redish and Touretzky, 1997; Hafting et al., 2005; O’Keefe and Burgess, 2005; Knierim et al., 2006; Witter and Moser, 2006; Gorchetchnikov and Grossberg, 2007; Kesner, 2007; Leutgeb and Leutgeb, 2007). Previous DG-manipulation studies (Lee et al., 2009, 2012b; Dees and Kesner, 2013; Morris et al., 2013) and the current study are consistent with this proposal. However, a recent study (Tang et al., 2014) has raised the possibility that layer II grid cells in the medial EC might be pyramidal neurons that do not project to DG/CA3, but rather project to CA1 (Kitamura et al., 2014). If this is the case, then the DG might have no access to the dead reckoning map. Contrary to this possibility, however, an *in vivo* intracellular recording study in mice engaged in a virtual navigation task has shown grid cell activity in both stellate and pyramidal cells in the medial EC layer II (Domnisoru et al., 2013). Moreover, a neural network simulation study has shown that physiological properties and connectivity of stellate cells can give rise to stable grid firing (Tamamaki and Nojyo, 1993; Couey et al., 2013). Clearly, additional studies are needed to clarify what type of information is provided from the EC to the DG and CA3. Nevertheless, previous empirical studies (Lee et al., 2009, 2012b; Dees and Kesner, 2013; Morris et al., 2013) and the current one provide evidence for a role of the DG in combining spatial and nonspatial information. Underlying neural mechanisms could be very different, however, depending on whether or not grid cells project to the DG.

A number of candidate functions have been proposed for the DG, and “pattern separation” in particular has garnered much attention, as well as empirical support (Kesner, 2007; Treves et al., 2008). In the present study, however, performance of Bax-KO mice was impaired at a 65 s delay even when the correct arm was the opposite of the sample arm, instead of an adjacent one, suggesting that behavioral impairment of Bax-KO mice was not due to reduced capability for spatial pattern separation (Gilbert et al., 2001; McHugh et al., 2007; Neunuebel and Knierim, 2014). It is worth noting that binding of spatial and nonspatial information and pattern separation are not incompatible functions of the DG. Pattern separation can also be achieved (or facilitated, at least) by conjunctive spatial and nonspatial coding (Kesner, 2007; Morris et al., 2013). Formation of a detailed spatial map of an environment and associating important events/objects with the map will allow fine discrimination of spatial locations where important events took place. In this regard, behavioral studies demonstrating impaired spatial discrimination following DG manipulation (Gilbert et al., 2001; Morris et al., 2012) might as well be explained by impaired spatial-nonspatial conjunctive coding. Likewise, impaired discrimination of similar contexts following DG manipulation (McHugh et al., 2007; Eadie et al., 2012) might also be explained by impaired spatial-nonspatial conjunctive coding. It would be difficult to discriminate two similar contexts solely based on nonspatial cues. Adding additional information that two contexts are located at two different places, which can be achieved by incorporating dead reckoning information in normal animals, might facilitate discrimination of two similar contexts. It remains to be determined whether the pattern separation function of the DG is a natural outcome of spatial-nonspatial conjunctive coding or represents a separate neural process.

It should be noted that we cannot completely rule out other deficits than impaired place-event association as the source for impaired performance of Bax-KO mice in the present task. Bax-KO mice might be impaired in remembering the temporal order of visited arms rather than place-event association. Because the animals were tested multiple times (20 days of training; six trials per day; total 120 trials per animal), they visited all arms multiple times over 20 days of training. If Bax-KO mice were impaired in discriminating between the time that a sample arm was visited (the arm visited in the current trial) and the time that an adjacent arm was visited (the arm that was visited a day ago, for example), then both will be recognized simply as previously visited arms. Although we cannot rule out this possibility, the finding that the hippocampus is not necessary for familiarity-based discrimination (Eichenbaum et al., 2007; Squire et al., 2007) argues against this possibility. Another possibility is that Bax-KO mice were impaired in processing spatial information (information on “where”) rather than place-event association. This is not likely, either, because the performance of Bax-KO mice was as good as that of wild-type mice in the Morris water maze task as long as goal locations predicted by dead reckoning and landmarks were congruent (Lee et al., 2009). Moreover, DG granule cells in Bax-KO mice showed location-specific discharges, albeit with lower spatial selectivity (Lee et al., 2009), indicating that the DG of Bax-KO mice represents spatial information. In addition, that Bax-KO mice were similarly impaired in the current task even when the correct arm was the opposite of the sample arm (day 20) also argues against impaired spatial information processing as the underlying cause for impaired performance of Bax-KO mice in the current task. As another possibility, the DG might process “what” information separately from spatial information in normal animals (with spatial-nonspatial conjunction occurring elsewhere, such as in CA3) and, in Bax-KO mice, “what” information may not be maintained when the delay is long (65 s) with an interruption. Although we cannot rule out this possibility, converging inputs from the lateral and medial EC to individual granule cells (Knierim et al., 2014; Moser et al., 2014) suggest that diverse types of information are integrated rather than processed separately in the DG. This possibility can be rejected if granule cells are found to concurrently code spatial and nonspatial information, as CA1 cells do (Wood et al., 1999; Frank et al., 2000; Ferbinteanu and Shapiro, 2003), which remains to be determined. Finally, we cannot rule out the possibility that the DG might be involved in transforming working memory into long-term memory regardless of its content (spatial or nonspatial of information). Information that exceeds working memory capacity might be stored temporarily in the DG before it is stored as a long-term memory, which remains to be tested.

## Author Contributions

WRK, JWL and MWJ designed the study; WRK and WS produced animal subjects; WRK, JWL and S-HL collected behavioral data; WRK, JWL, J-SC and MWJ analyzed the data; JWL and MWJ wrote the manuscript with inputs from all other authors.

## Conflict of Interest Statement

The authors declare that the research was conducted in the absence of any commercial or financial relationships that could be construed as a potential conflict of interest.
